# Separase Inhibitor Sepin-1 Inhibits Foxm1 Expression and Breast Cancer Cell Growth

**DOI:** 10.4172/1948-5956.1000517

**Published:** 2018-03-22

**Authors:** Nenggang Zhang, Debananda Pati

**Affiliations:** Department of Pediatrics, Texas Children’s Cancer Center, Baylor College of Medicine, Houston, Texas, USA

**Keywords:** Separase, Sepin-1, Raf, FoxM1, Cell proliferation, Breast cancer

## Abstract

Sepin-1, a potent non-competitive inhibitor of separase, inhibits cancer cell growth, but the mechanisms of Sepin-1-mediated growth inhibition are not fully understood. Here we report that Sepin-1 hinders growth of breast cancer cells, cell migration, and wound healing. Inhibition of cell growth induced by Sepin-1 *in vitro* doesn’t appear to be through apoptosis but rather due to growth inhibition. Following Sepin-1 treatment caspases 3 and 7 are not activated and Poly (ADP-ribose) polymerase (Parp) is not cleaved. The expression of Forkhead box protein M1 (FoxM1), a transcription factor, and its target genes in the cell cycle, including Plk1, Cdk1, Aurora A, and Lamin B1, are reduced in a Sepin-1-dependent manner. Expressions of Raf kinase family members A-Raf, B-Raf, and C-Raf also are inhibited following treatment with Sepin-1. Raf is an intermediator in the Raf-Mek-Erk signaling pathway that phosphorylates FoxM1. Activated FoxM1 can promote its own transcription via a positive feedback loop. Sepin-1-induced downregulation of Raf and FoxM1 may inhibit expression of cell cycle-driving genes, resulting in inhibition of cell growth.

## Introduction

Separase is a sister chromatid cohesion-resolving enzyme that cleaves cohesin subunit Rad21, resulting in the segregation of sister chromatids during cell cycle [1]. Besides its canonical role, separase is required for centrosome duplication [2–6], DNA damage repair [7,8] and membrane trafficking [9–11]. Because of its involvement in numerous important cell processes, separase is tightly regulated in normal cells [12–16]. However, separase has been found to be an oncogene and overexpressed in many human cancers, including breast, bone, brain, and prostate tumors [17–21]. Overexpression of separase promotes aneuploidy, genomic instability, and tumorigenesis in mouse models [19,20].

In order to modulate the activity of Separase in cancer cells, we have performed a high throughput screen and identified a small molecular inhibitor called Sepin-1 [22]. Sepin-1 non-competitively inhibits separase activity and prevents the growth of leukemia, neuroblastoma, and breast cancer cells *in vitro*, as well as breast-cancer tumors *in vivo* in a separase-dependent manner [22]. However, the mechanisms behind the oncostatic action of Sepin-1 have not been elucidated. Herein we report that Sepin-1 inhibits breast-cancer cell growth, migration, and wound healing in a dose-dependent manner. The inhibition is not through apoptosis but likely through the inhibition of FoxM1, an important cell cycle transcription factor, and its target gene expression.

## Materials and Methods

### Antibodies

Cdk1 mouse antibody (mAb) (610037) and PARP mAb (7D3-6) from BD Biosciences (San Jose, CA); Lamin B1 rabbit antibody (rAb) (ab16048), Caspase 3 rAb (ab32351), and Pericentrin rAb (ab4448) from Abcam (Cambridge, MA); Caspase 7 mAb (05-578) from Upstate Biotechnology, Inc. (Lake Placid, NY); Plk1 mAb (37-7100) from Thermo Fisher Scientific (Waltham, MA); Aurora A mAb (MCA2249) from Bio-Rad (Hercules, CA); A-Raf rAb (4432), B-Raf rAb (9433), and C-Raf mAb (12552) from Cell Signaling Technologies (Danvers, MA); GAPDH mAb (GTX627408) from GeneTex (Irvine, CA); β-Actin mAb (A5316) from Sigma-Aldrich (St. Louis, MO).

### Drugs

Sepin-1 was synthesized by ChemBridge (San Diego, CA); Etoposide was from Sigma-Aldrich (St. Louis, MO).

### Tissue culture

BT-474, MCF7, MDA-MB-231, and MDA-MB-468 were obtained from American Type Culture Collection (ATCC) (Manassas, VA). The cells were cultured according to the protocols of ATCC.

### qPCR

One million cells were seeded to a 10cm plate overnight and treated with different concentrations of Sepin-1 for 24 h. Total RNA was extracted using RNeasy Plus Mini Kit (Qiagen, Inc, Valencia, CA). cDNA was synthesized using RT^2^ First Strand Kit (Qiagen), and qPCR was performed using RT^2^ SYBR Green ROX qPCR Mastermix (Qiagen) in Eppendorf Mastercycler Realplex4.

### Cell-migration assay

Cell migration following treatment with Sepin-1 was assayed using the protocol reported previously with certain modifications [23]. Briefly, cells were detached and suspended in serum-free cell culture media with 1×10^5^ cells/ml. One hundred μl of the cell solution was plated on the top of the filter membrane in a 24-well Transwell^®^ insert (Corning) and incubated for 10 minutes at 37°C to allow the cells to settle down. Six hundred μl of medium with or without Sepin-1 was added to the bottom of the lower chamber in a 24-well plate. The cells were incubated at 37°C for 24 h. To examine the cells that migrated to the other side of the membrane, the cells that did not migrate were first removed using cotton-tipped applicators. The cells on the opposite side of the membrane on the Transwell^®^ insert were fixed with 4% glutaraldehyde and stained with 0.2% crystal violet. The migrated cells were counted with an inverted microscope (AXIO Vert. A1, Zeiss). Five random fields of each membrane were examined. The number of cells in those fields were averaged and represented as the cells that migrated. The assay was performed in triplicate.

### Wound healing assay

The wound healing assay was performed according to the protocol reported previously [23]. Briefly, 2 × 10^5^ cells per well were seeded to a 12-well plate. After 24h incubation at 37°C, a straight line of wound was made by using a 200 μl pipette tip in each well. The medium was carefully aspirated to remove the cell debris and replaced with medium containing with or without Sepin-1. To check for wound closure, images were taken at 0, 24, 48, and 72 h after the wound was made. The distance of the wound gap was measured using a scale bar.

### TUNEL assay

Cells were seeded to 6-well plates and treated with Sepin-1 for 24 h. The cells were detached and cytospun to slides. The cells were then fixed, permeablized, and labeled by following the protocol using DeadEnd^™^ Fluorometric TUNEL System (Promega, Madison, WI). The slides were examined under a fluorescence microscope.

### Reverse phase protein array (RPPA)

Cells were treated with Sepin-1 for 24 h, and whole cell lysate was prepared as reported previously [24]. RPPA was performed by RPPA Core Facility at MD Anderson Cancer Center, Houston, TX.

### Immunoblotting

Protein preparation and Western blot were performed as reported previously [24,25].

### Cell viability assay

Cells were seeded to 96-well plates in 100 μl medium per well and incubated at 37°C for 24 h. On the second day, 50 μl of Sepin-1 or medium was added to each well. On the fifth day, 20 μl of CellTiter-Blue® Reagent (Promega) was added to each well. After 6h incubation, the fluorescence intensity (FI) was determined using a microplate reader at excitation 560nm and emission 590nm. The wells without cells served as background, and the wells without Sepin-1 treatment served as the positive control. Cell viability (%)=100 × ((FI_Sepin-1_ − FI_background_)/(FI_positive_ − FI_background_). Each treatment was performed in triplicate and repeated with 3 independent experiments.

## Results

### Sepin-1 inhibits growth of breast cancer cells

**Figure 1:**
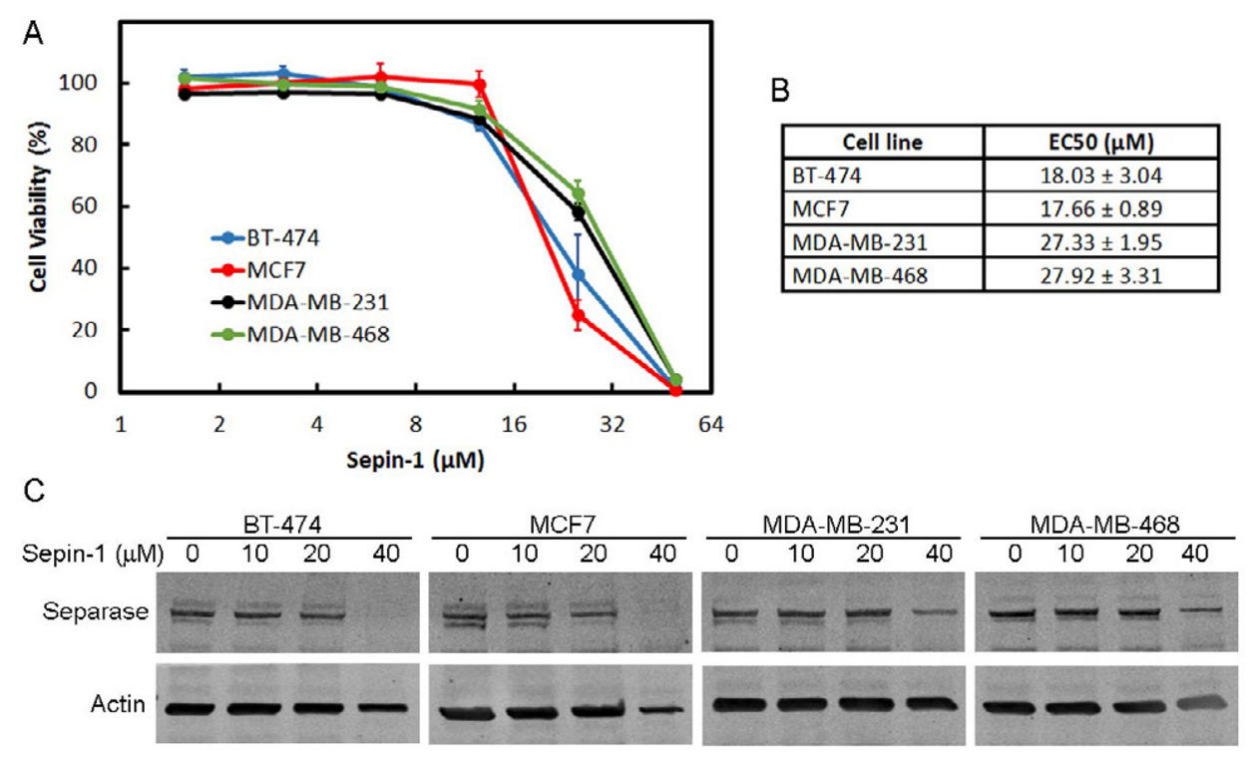
Sepin-1 inhibits growth of breast cancer cells. Breast cancer cell lines were seeded to 96-well plates overnight and treated with various concentrations of Sepin-1 for 3 days. The viability of cells was determined using CellTiter-Blue® Reagent. Three independent experiments were conducted. (A). Cell viability curve of breast cancer cells. (B). The concentration of Sepin-1 that inhibited 50% of the cells. (n=3 ± SD). (C). Breast cancer cells were plated and treated with Sepin-1 for 24 h before protein samples were made. Immunoblotting was performed and probed with indicated antibodies.

Four breast cancer cell lines, BT-474, MCF7, MDA-MB-231, and MDA-MB-468, with distinct hormone-receptor status and subtypes (Table 1) were used for these studies. Sepin-1 inhibited the cell growth of all four cell lines (Figure 1A). BT-474 and MCF7 (EC_50_=~18 μM) were more sensitive than triple-negative cell lines MDA-MB-231 and MDA-MB-468 (EC_50_=~28 μM) (Figure 1B). Immunoblotting results showed that separase level was reduced when cells were treated with Sepin-1 (Figure 1C), and the reduction of separase was more significant in BT-474 and MCF7 cells than that in the two triple negative cell lines (Figure 1C).

### Sepin-1 inhibits cell migration and wound healing

**Figure 2:**
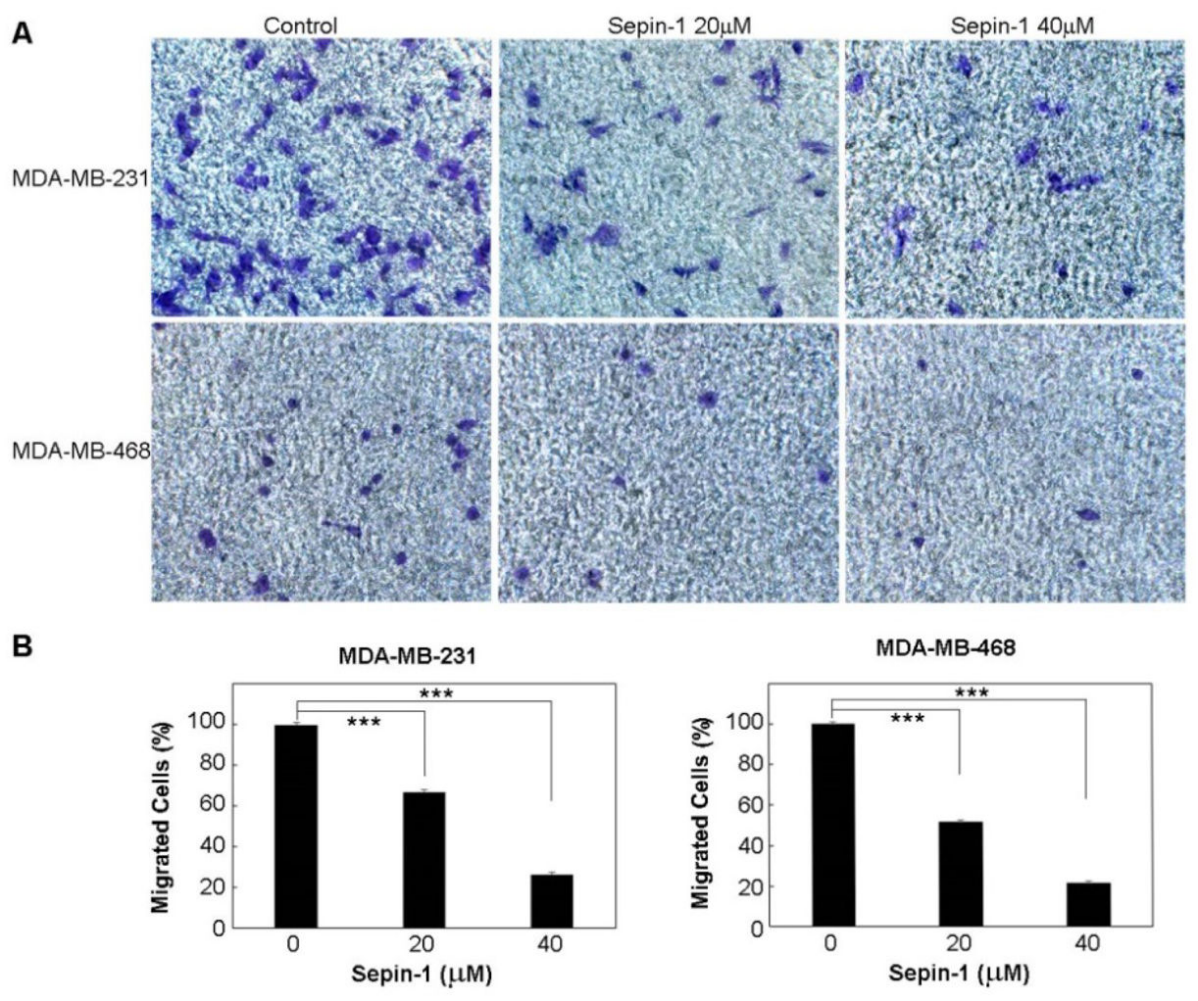
Sepin-1 inhibits cell migration of triple-negative breast cancer cells. MD-MB-231 or MD-MB-468 cells were seeded on the top of the filter membrane in a Transwell^®^ insert, and medium with or without Sepin-1 was added to the bottom of the lower chamber in a 24-well plate. After 24 h of incubation, the cells that had not migrated were removed. The cells on the other side of the membrane on Transwell^®^ insert were fixed and stained with crystal violet. The migrated cells (in purple) were counted. Representative images of migrated cells are shown in A. Percentage of cells migrated through the membrane is shown in B. The assay was performed in triplicate (n=3 ± SD). *** indicates p<0.05.

Metastasis occurs more frequently in basal breast carcinoma than in luminal breast cancers. Cell migration assay commonly is used to determine the potential capability for metastasis *in vitro.* We tested the efficacy of Sepin-1 in inhibiting cell migration using both the luminal and basal cell lines. We did not find any migration of cells for the two luminal breast cancer cell lines, BT-474 and MCF7, whereas the two basal breast cancer cell lines, MDA-MB-231 and MDA-MB-468, could easily migrate through the membrane of the Transwell^®^ (Figure 2A). The migration was significantly inhibited by Sepin-1 (Figure 2B).

**Figure 3:**
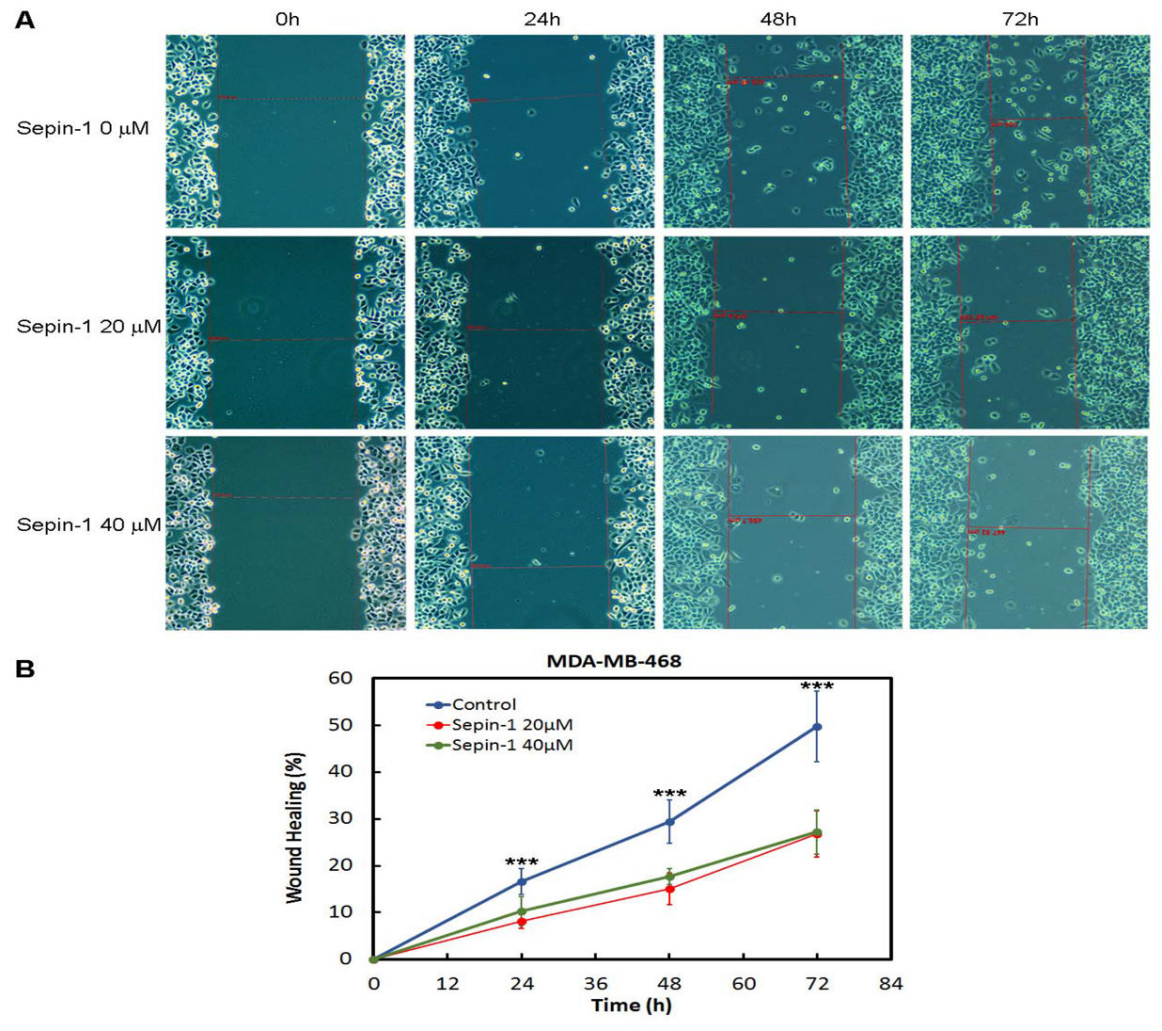
Sepin-1 inhibits wound healing of MDA-MB-468 cells. MD-MB-468 cells were grown on 12-well plates, and a straight line of wound was made. The medium was carefully aspirated to remove the cell debris and replaced with medium with or without Sepin-1. To check for wound closure, images were taken at 0, 24, 48 and 72 h after the wound was made. The distance of the wound gap was measured using a scale bar as shown in (A) with red lines. Representative images of migrated cells are shown in A. The percentage of wound gap closure is shown in (B). The assay was performed in triplicate (n=3 ± SD). *** indicates p<0.05.

As an additional test, we confirmed the inhibitory effect of Sepin-1 on cell migration using a wound healing assay. Sepin-1 significantly reduced wound healing of MDA-MB-468 cells (Figures 3A and 3B). The closure of the wounds’ gaps in control groups occurred significantly faster than that of the Sepin-1-treated groups, which could be seen as early as 24 h after treatment with Sepin-1. There was no significant difference between 20 μM and 40 μM Sepin-1-treated groups (Figure 3B).

### Sepin-1 causes DNA damage in BT-474 and MCF7 cell lines

**Figure 4:**
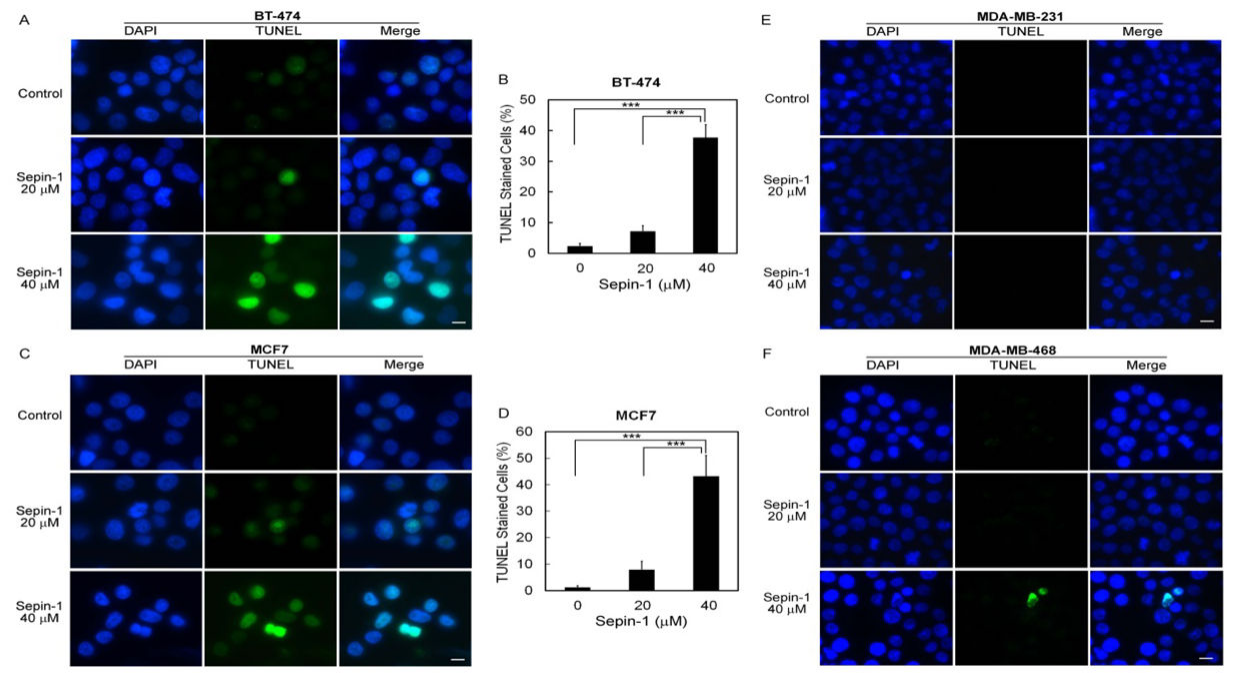
TUNEL assay in breast cancer cells after treatment with Sepin-1. Cells were seeded to 6-well plates and treated with 0, 20, and 40 μM of Sepin-1 for 24 h. The cells were detached and cytospun to slides. The samples were fixed, permeablized, and labeled with DeadEnd^™^ Fluorometric TUNEL System (Promega). Images were taken from seven random fields, and TUNEL-positive cells were counted and the percentage was calculated for BT-474 (A, B) and MCF7 (C, D). Almost no TUNLE-positive cells were found in MDA-MB-231 (E) and MDA-MB-468 (F). *** indicates p<0.05. Scale bar, 10μm.

To investigate whether or not Sepin-1 treatment can cause DNA fragmentation, we performed terminal deoxynucleotidyl transferase (TdT) dUTP Nick-End Labeling (TUNEL) assay. According to our results, approximately 40% of both BT-474 and MCF7 cells treated with 40μM Sepin-1 were TUNEL-positive. This percentage was significantly higher than that in control cells or cells with 20μM Sepin-1 treatment, which was less than 10% (Figures 4A–4C). Surprisingly, we seldom found TUNEL-positive staining in MDA-MB-231 and MDA-MB-468 cells treated with 40μM Sepin-1 (Figures 4E and 4F). We have excluded the possible technical issues by performing the experiments side-by-side. We speculate that luminal and basal breast cancer cells likely have different responses to Sepin-1 treatment.

### Cell growth inhibition following Sepin-1 treatment is not through apoptosis

**Figure 5:**
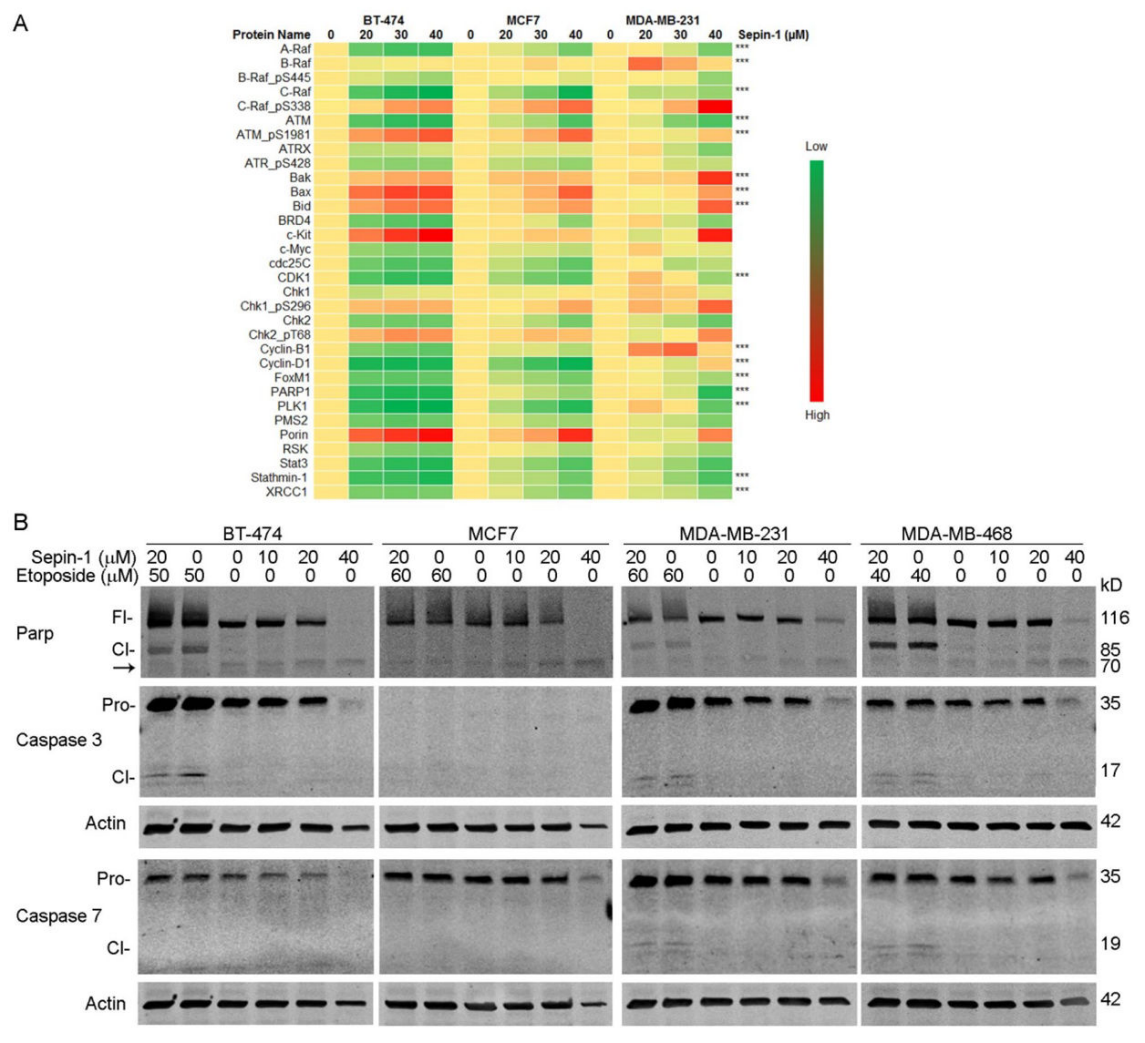
Sepin-1 inhibits growth of breast cancer cells but not via apoptosis. (A). Breast cancer cells were treated with Spein-1 for 24 h. Protein samples were made and used for Reverse Phase Protein Array (RPPA) assay. *** indicates the result was verified in immunoblotting. (B). Breast cancer cells were plated and treated with Sepin-1 with or without Etoposide for 24 h before protein samples were made. Immobloting was performed with indicated antibodies. MCF7 does not have caspase 3. Fl, full length; Cl, cleaved; Pro, proenzyme; arrow, unknown cleaved product.

One of the mechanisms that lead to cell death is apoptosis. Many proteins are involved in apoptotic signaling cascade. Results from Reverse Phase Protein Array (RPPA) assay indicated that pro-apoptotic regulators Bak, Bax, and Bid increased in a Sepin-1 concentration-dependent manner (Figure 5A), suggesting that an apoptotic signaling cascade was initiated. We expected that effector enzymes caspases 3 and 7 would be activated with the generation of the 17 kD and 19 kD fragments, respectively, and Parp would be cleaved with the generation of the 85kD fragment after cells were treated with Sepin-1. To our surprise, in Sepin-1-treated samples, we did not find the cleaved fragments of activated caspases 3 and 7 or cleaved 85 kD Parp (Figure 5B). Etoposide can induce apoptosis and was used as the positive control. Immunoblotting results indicated that treatment with Etoposide caused cleavage of Parp, resulting in the generation of an 85 kD fragment in BT-474, MDA-MB-231, and MDA-MB-468 cells (Figure 5B). In those three cell lines, the proenzymes of caspase 3 were also activated with the generation of cleaved fragments (17 kD), whereas caspase 7 was activated (19 kD) only in MDA-MB-231 and MDA-MB-468 but not in BT-474 or MCF7 (Figure 5B). No caspase 3 bands were found on MCF7 blot because MCF7 is known to be caspase 3 deficient. Apoptotic Parp cleavage (85 kD fragment) was not found in MCF7 (Figure 5B), which is possibly due to lack of activated caspases 3 and 7.

RPPA data showed that Parp protein levels were reduced in cells treated (Figure 5A), which was confirmed in immunoblotting (Figure 5B). The reduction of Parp possibly is not caused by apoptosis because the 85 kD fragment of Parp was not found, and caspases 3 and 7 were not activated (Figure 5B). However, we noted a faint band at approximately 70kD (Figure 5B arrow), possibly a degraded fragment of Parp that was resulted from the treatment with Sepin-1.

### Sepin-1 inhibits expression of Raf and FoxM1

**Figure 6:**
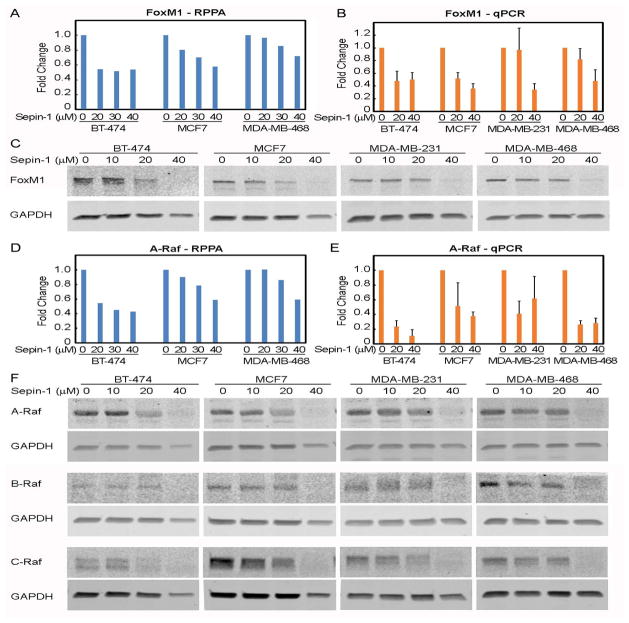
Sepin-1 reduces expression of FoxM1. Breast cancer cells were treated with Spein-1 for 24 h. Protein samples were made and used for RPPA (A, D) and immunoblotting (C, F). Total RNA was prepared and used for qPCR (B, E). Three independent experiments were performed for immunoblotting and qPCR. One representative immunoblotting is shown. qPCR results were average of three experiments (n=3 ± SE).

Because Sepin-1 inhibits cell growth not via apoptosis, we hypothesized that the inhibition is possibly through its effect on cell proliferation. We performed a reverse phase protein array (RPPA) assay and found that expression of FoxM1 was significantly reduced following treatment with Sepin-1 (Figure 6A). qPCR analysis further indicated that mRNA of FoxM1 was reduced (Figure 6B), and immunoblotting data confirmed the RPPA results showing that levels of FoxM1 protein were decreased in cells treated with Sepin-1 (Figure 6C).

FoxM1 is a transcription factor, and Raf-MEK-Erk signaling cascade plays an important role in its activation. RPPA and qPCR results showed that A-Raf expression was inhibited following treatment with Sepin-1 (Figures 6D and 6E). Western blot data also confirmed that expressions of A-Raf, B-Raf, and C-Raf were inhibited by Sepin-1 (Figure 6F).

### Sepin-1 inhibits the expression of proliferation-promoting proteins

**Figure 7:**
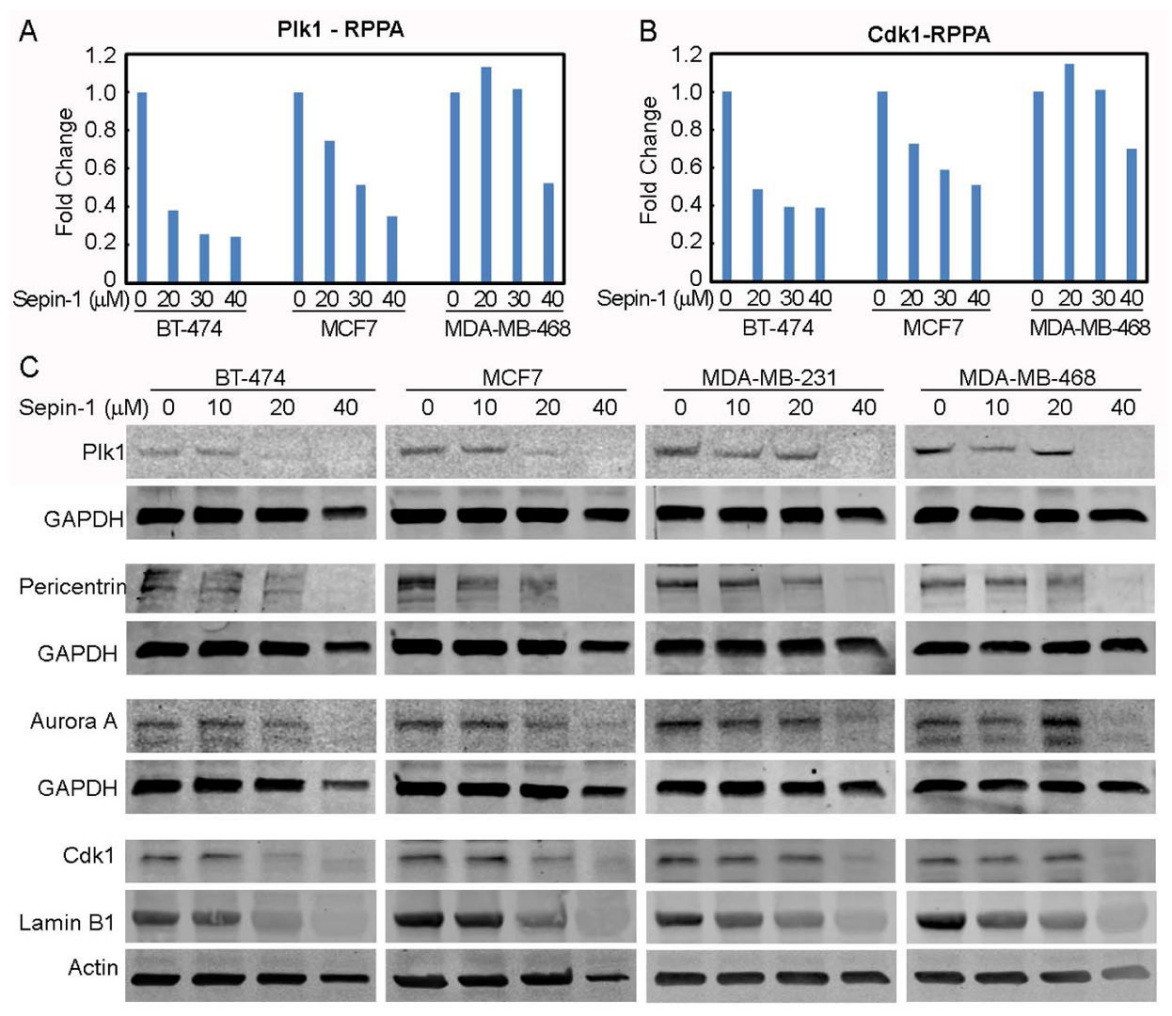
Spein-1 reduces the levels of cell cycle-driving proteins. Breast cancer cells were treated with Spein-1 for 24 h before protein samples were made and used for RPPA (A, B) and immunoblotting (C). A-B. RPPA assay of Plk1 and Cdk1. C. Immunoblotting of Plk1, pericentrin, Aurora A, Cdk1, and Lamin B1. GAPDH and actin were used as a loading control.

Many FoxM1-targeting proteins control cell cycle progression and proliferation. Plk1, Cdk1, Aurora A, pericentrin, and Lamin B1 are among those proteins. RPPA data and Western blot showed that Sepin-1 reduced Plk1 and Cdk1 protein levels (Figures 7A–7C). Pericentrin, Aurora, and Lamin B1 were also inhibited by Sepin-1 (Figure 7C).

## Discussion

Separase is overexpressed in many cancers, including breast cancer tumors [1,17,18,20,22,26,27]. Sepin-1 is a separase inhibitor and inhibits growth of cancer cells [22]. Although Sepin-1 can induce apoptosis in Molt4 cells [22], it is unclear how Sepin-1 inhibits cell growth in breast cancer cells. Among the breast cancer cell lines that we used in this study, BT-474 and MCF7 are classified to luminal B and luminal A subtype breast cancer, respectively. MDA-MB-231 and MDA-MB-468 are basal-like breast cancer cells. Although the cell growth of all four cell lines was inhibited by Sepin-1, their responses to Sepin-1 were different in some degree. Luminal breast cancer cell lines BT-474 and MCF7 were more sensitive to Sepin-1 than were the basal breast cancer cell lines MDA-MB-231 and MDA-MB-468 (EC50=~18 vs ~28 μM). DNA damage was obvious in luminal breast cancer cell lines treated with Sepin-1 but not in basal-like breast cancer cell lines. Metastasis is defined as cancer cells that migrate to a location different from the origin. It is well-known that MDA-MB-231 and MDA-MB-468 cell lines are metastatic. Sepin-1 could inhibit migration and wound healing in MDA-MB-231 and MDA-MB-468. The effects of treatment with Sepin-1 that are similar on the four breast cancer cell lines include non-activation of caspases 3 and 7 and downregulation of cell cycle-driving proteins, such as Raf, FoxM1, Plk1, Cdk1, Aurora A, Lamin B1, and pericentrin.

Sepin-1 inhibits growth of breast cancer cells, but we do not know how. Growth of a tumor is determined by cell proliferation and cell death. Inhibition of cell proliferation and/or promotion of cell death is necessary to inhibit a tumor’s growth. Many factors lead to cell death [28,29]. We explored the possibility of apoptosis induced by Sepin-1. Activation of effector caspases 3 and 7 and Parp cleavage are the hallmarks of apoptosis. RPPA assay indicated that pro-apoptotic regulators Bak, Bax, and Bid increased in a Sepin-1, concentration-dependent manner, suggesting that an apoptotic signaling cascade was initiated. However, contrasting to the apoptotic positive control of cells treated with etoposide, neither caspase 3 nor caspase 7 was activated, nor was Parp cleaved in Sepin-1-treated cells, suggesting that treatment with Sepin-1 hinders execution of apoptosis in breast cancer cell lines.

Cell proliferation requires expression of cell cycle driving factors, such as FoxM1, Plk1, Cyclin B1, Cdk1, Lamin B1, and pericentrin. All are target genes regulated by FoxM1 transcription factor [30], and their expressions are inhibited by Sepin-1. An interesting note is that a strong positive correlation exists between ESPL1 (gene that encodes Separase protein) and FoxM1 transcripts across various human tumor tissues, suggesting a relationship between FoxM1 and Separase expression in human cancer. Forkhead box (Fox) proteins are evolutionarily conserved transcription factors that consist of a superfamily with 49 members that are classified into 19 subfamilies [31]. FoxM1 is one of the subfamilies and has four isoforms, FoxM1a, FoxM1b, FoxM1c, and FoxM1d [32,33]. FoxM1 regulates cell cycle progression, proliferation, migration, metabolism, DNA damage response, angiogenesis, metastasis, and tumor development and progression [34].

The activity of FoxM1 can be affected in several ways. First, the expression of FoxM1 is self-regulated because it possesses a positive feedback loop and activates its own transcription [35,36]. Next, the degradation of FoxM1 can be regulated through proteasome. Thiazole antibiotics thiostrepton and siomycin A inhibit FoxM1 expression and its transcriptional activity [37–39]. It has been proposed that thiostrepton, siomycin A, and other proteasome inhibitors hinder degradation of a negative regulator of FoxM1, which in turn reduces the activity of FoxM1 [36,40–42]. Furthermore, the activity of FoxM1 is regulated by post-translation modifications. Phosphorylation by the Ras-Raf-Mek-Erk cascade leads to FoxM1 nuclear translocation. Once inside the nucleus, FoxM1 is activated through phosphorylation by Cdk1/cyclin B1 and Plk1 to promote transcription of Cdk1, cyclin B1, Plk1 in a positive feedback loop that produces a burst of Cdk1 and Plk1 activity to drive the cells into mitosis [43,44]. Inhibition of cell growth by Sepin-1 could be achieved through attenuating this positive feedback loop.

The Raf kinase family has three members, A-Raf, B-Raf, and C-Raf/Raf-1. The well-known role of Rafs is in Ras pathway signaling, in which they function as a direct effector of the Ras GTPases and as the initiating kinase in the Mek-Erk cascade [45]. Raf contains a Ras-binding domain (RBD) in the N-terminal regulatory region, which interacts to active GTP-bound Ras [46]. This interaction disrupts auto-inhibition and induces phosphorylation and dimerization of Raf. Raf can form heterodimer with other Raf family members, which is required for the activation of Raf kinase [47–50]. Catalytically active Raf kinase phosphorylates and activates Mek, which in turn phosphorylates and activates Erk [46]. After phosphorylated by Erk, FoxM1 is translocated to the nucleus and binds to the promoter region of its target genes to stimulate their transcription [44]. Raf proteins are reduced in cells treated with Sepin-1, which could reduce the activity of the Ras-Raf-Mek-Erk cascade, in turn decreasing the phosphorylation and nuclear translocation of FoxM1. Regulation of Raf kinase expression by FoxM1 has not been reported. The mechanisms of how Sepin-1 downregulates Raf remain to be elucidated.

## Conclusion

In summary, Sepin-1 inhibits breast cancer cell growth not via apoptosis but possibly via inhibition of cell proliferation. We speculate that inhibition by Sepin-1 of Raf expression negatively affects the phosphorylation of FoxM1 by the Raf-Mek-Erk pathway, and possibly its nuclear translocation. Reduced FoxM1 decreases the expression of FoxM1 itself and causes the collapse of FoxM1 expressing positive feedback loop, ultimately leading to the inhibition of FoxM1 target genes’ expressions, which are critical to the progression of cell-cycle and the proliferation of cells.

## Figures and Tables

**Table 1:** The molecular classification of the four breast cancer cell lines used in this study.

Cell Line	Subtype	ER	PR	Her2
BT-474	Luminal B	+	+	+
MCF7	Luminal A	+	+	−
MDA-MB-231	Basal-like	−	−	−
MDA-MB-468	Basal-like	−	−	−
